# circ_0001588 Induces the Malignant Progression of Hepatocellular Carcinoma by Modulating miR-874/CDK4 Signaling

**DOI:** 10.1155/2021/3759879

**Published:** 2021-10-20

**Authors:** Xiaoyun Bin, Yichen Chen, Jiasheng Ma, Renchao Tang, Zhenrong Zhao, Kangxuan Wang, Jianchu Wang

**Affiliations:** ^1^College of Basic Medical Sciences, Youjiang Medical University for Nationalities, Baise, China; ^2^Department of Hepatobiliary Surgery, Affiliated Hospital of Youjiang Medical University for Nationalities, Baise, China

## Abstract

Accumulating evidence indicates that circular RNAs (circRNAs) can interact with microRNAs to modulate gene expression in various cancers, including hepatocellular carcinoma (HCC). Although the significant role of circRNAs has been well documented in HCC, the complex mechanisms of circRNAs still need to be elucidated. Our current study is aimed at investigating the function of circ_0001588 in HCC, which was observed to significantly increase in HCC tissues and cells. We demonstrated that the knockdown of circ_0001588 resulted in repressed cell proliferation, migration, and invasion. *In vivo* studies using a nude mouse model showed that circ_0001588 downregulation reduced tumor size. Moreover, miR-874 was predicted as a target of circ_0001588. Using luciferase binding assays, we proved that circ_0001588 functions as a molecular ceRNA of miR-874 and that CDK4 acts as a downstream target of miR-874 in HCC. It was confirmed that overexpression of miR-874 decreased the proliferation, migration, and invasion triggered by the increase in circ_0001588. In summary, our results indicate that circ_0001588 acts as a ceRNA and promotes HCC progression by targeting the miR-874/CDK4 signaling pathway. Hence, we propose that circ_0001588 may be a promising target for HCC treatment.

## 1. Introduction

Hepatocellular carcinoma (HCC) accounts for 90% of primary liver carcinomas worldwide [1, 2] and is a leading cause of malignant tumors in humans with high morbidity and mortality rates [3, 4]. Although great advances have been made in the therapeutic strategies for HCC treatment, attempts to increase the average 5-year survival rate of HCC patients remain unsatisfactory [5]. To date, little is known about the pathogenesis of HCC. Hence, it is crucial to understand the pathology of HCC and identify the underlying mechanisms.

Circular RNAs (circRNAs) have been reported to play significant roles in various cell activities [6–8]. Several studies have shown that circRNAs can regulate multiple tumor processes such as cell proliferation, apoptosis, migration, and invasion [9–11]. For example, circRNA BCRC-3 can repress bladder cancer development by regulating miR-182-5p and p27 [12]. circ_100395 regulates miR-1228 and TCF21 to inhibit lung cancer development [13], and circRNA UBAP2 can induce ovarian cancer progression by sponging miR-144 [14].

circRNAs are important biomarkers for HCC. It has been reported that circRNAs can demonstrate oncogenic or suppressive effects on HCC development by regulating various biological processes [15, 16]. For example, circRHOT1 promotes HCC progression through the initiation of NR2F6 [17]. circTRIM33-12 serves as a sponge for miR-191 to repress HCC progression [18]. circ_0001588, located on chromosome 6, has been reported to be abnormally highly expressed in lung adenocarcinoma [19]. However, its biological role and molecular mechanisms in HCC remain poorly understood.

In this study, we aimed to investigate the mechanism of circ_0001588 in contributing to the malignant phenotypes of HCC.

## 2. Materials and Methods

### 2.1. Cell Culture

Human HCC cell lines used in this study (SK-Hep-1, Hep-3B, HepG2, BEL-7402, and MHCC-LM3) and the control hepatic normal cell line (LO2) were obtained from the Shanghai Institute of Cell Biology, Chinese Academy of Sciences (Shanghai, China). Cells were incubated in DMEM medium (Gibco, Grand Island, NY, USA) supplemented with 10% heat-inactivated FBS, 1% penicillin (100 U/mL), and 0.1 mg/mL streptomycin in a humidified chamber with 5% CO_2_ and 95% air at 37°C.

### 2.2. Plasmid Construction and Cell Transfection

To overexpress circ_0001588, the full-length circ_0001588 cDNA was amplified in HCC cells and then cloned into the overexpression vector pLCDH-ciR (Genechem, Shanghai, China). All plasmids were isolated using a DNA Midiprep kit (Qiagen, Hilden, Germany). To perform miR-874 overexpression and knockdown, miR-874 mimics, miR-874 inhibitor, and two scrambled miRNAs used as negative controls were purchased (Genechem). Transfections were performed using the Lipofectamine® 3000 reagent (Invitrogen, Waltham, MA, USA).

### 2.3. CCK-8 Assay

The transfected cells were seeded into 96-well plates at a density of 1 × 10^4^ cells per well. A Cell Counting Kit-8 (A311-01, Vazyme, Nanjing, China) was used to detect cell viability. Absorbance was measured at 450 nm using a microplate reader.

### 2.4. Detection of Apoptosis and Cell Cycle

Cell apoptosis and cell cycle phase distribution indicated by cellular DNA content were analyzed by flow cytometry. HCC cells were harvested, washed with cold PBS, fixed in 70% ethanol, and treated with 10 mg/mL RNase A. Fixed cells were stained with PI dye followed by incubation in the dark. The PI fluorescence of individual nuclei was measured using a flow cytometer (BD FACSCalibur, Becton Dickinson, Franklin Lakes, NJ, USA). The data were analyzed using Cell Quest Pro V 3.2.1 software (Becton Dickinson).

### 2.5. Transwell Migration and Invasion Assay

To carry out the invasion experiment, Matrigel was diluted to 1 mg/mL on ice using a precooled serum-free medium; 40 *μ*L per well was added to a Transwell chamber. Then, 100 *μ*L and 600 *μ*L of serum-free medium were added to the upper and lower chambers, respectively, and equilibrated overnight at 37°C. 100 *μ*L of the cell suspension and 600 *μ*L of complete medium were added to the Transwell chamber. The upper chamber was placed in a culture well. After incubation for 48 h, the upper chamber was removed. Crystal violet (0.1% concentration; 500 *μ*L) was added to a 24-well plate. The chamber was placed, and the membrane was immersed in the medium at 37°C for 30 min. Finally, five fields of view diameters were selected, imaged, and counted. To carry out the migration experiment, the cells were incubated in the Transwell chamber for 24 h. The other steps were the same as those used for the invasion assay.

### 2.6. Western Blotting

Cells were washed and proteins were extracted using lysis buffer (100 *μ*L/50 mL). Protein samples were separated using SDS-PAGE and transferred to polyvinylidene fluoride membranes (Millipore, Burlington, MA, USA). Membranes were incubated with anti-CDK4 and GAPDH primary antibodies (Abcam, Cambridge, UK). The next day, the membranes were incubated at room temperature with a horseradish peroxidase-labeled secondary antibody (Santa Cruz Technology, Santa Cruz, CA, USA) for 1 h. Membranes were visualized using an enhanced chemiluminescence (ECL) system.

### 2.7. RT-PCR

Total RNA was extracted using the TRIzol reagent (Invitrogen). For miRNA analysis, cDNA was obtained using the TaqMan MicroRNA Reverse Transcription Kit (Applied Biosystems, Waltham, MA, USA). qRT-PCR was conducted using the TaqMan miRNA assay kit (Applied Biosystems). For mRNA analysis, cDNA was synthesized using M-MLV reverse transcriptase (Invitrogen) and reverse transcription primer Oligo(dT). Then, qRT-PCR was carried out using SYBR Green Real-Time PCR Master Mix (Thermo Fisher Scientific, Waltham, MA, USA) on a 7900HT Fast Real-Time PCR machine (Applied Biosystems). The primers used are listed in Table 1.

### 2.8. Luciferase Reporter Analysis

Cells were seeded in 24-well plates overnight. Luciferase reporters including circ_0001588-WT, circ_0001588-MUT, CDK4-WT, and CDK4-MUT were constructed by Genechem. A QuikChange Lightning kit (Stratagene, La Jolla, CA, USA) was used to perform site-directed mutagenesis. Sequencing was performed to confirm the correct mutations. Luciferase activity was measured using the dual-luciferase reporter assay system (Promega, Madison, WI, USA).

### 2.9. RNA Immunoprecipitation Assay

Cells (~1 × 10^7^) were washed with cold PBS and lysed with RIP lysis buffer (EMD Millipore). Cell lysates were incubated with RIP immunoprecipitation buffer containing magnetic beads conjugated with human Ago2 antibody (Millipore) or negative control mouse IgG (Millipore). Extracted RNAs were analyzed using qRT-PCR to test for the presence of circ_0001588 and miR-874.

### 2.10. Animal Studies

Twelve BALB/c, all-female nude mice (6–8 weeks old) were obtained from the Chinese Science Academy (Shanghai, China). Mice were injected with 5 × 10^6^ WT HepG2 cells (control) or circ_0001588 shRNA-transfected HepG2 cells (12 nude mice were randomly divided into 2 groups, 6 in each group). The growth of implanted HCC tumors was monitored for their volumes every 5 days up to 22 days postinoculation. Xenograft tumor size was recorded every three days (volume = width^2^ × length × 1/2). Tumors were fixed in 10% formalin, embedded in paraffin, and cut into 4 *μ*m thick slices. Hematoxylin and eosin staining was used to observe the tumor histology. Animal procedures were performed according to the *Guide for the Care and Use of Laboratory Animals* of the National Institutes of Health. The assays were approved by the Animal Care and Use Committee of the Affiliated Hospital of Youjiang Medical University for Nationalities.

### 2.11. Flow Cytometry of Ki-67 Analysis

To test the frequencies of Ki-67 positive cells, tumor cells were fixed, permeabilized, and stained with anti-Ki-67-FITC (BD Biosciences, Franklin Lakes, NJ, USA). Cells stained with fluorophore-conjugated secondary antibodies were analyzed by flow cytometry.

### 2.12. Statistical Analysis

Data were analyzed using the SPSS software (version 19.0). All experiments were conducted in triplicate, and the values are presented as the mean ± SEM. Two-tailed Student's *t*-test was used to analyze the statistical differences between the two groups. Multiple groups were compared using a one-way analysis of variance. Statistical significance was set at *P* < 0.05.

## 3. Results

### 3.1. circ_0001588 Is Upregulated in HCC Cells

**Figure 1:**
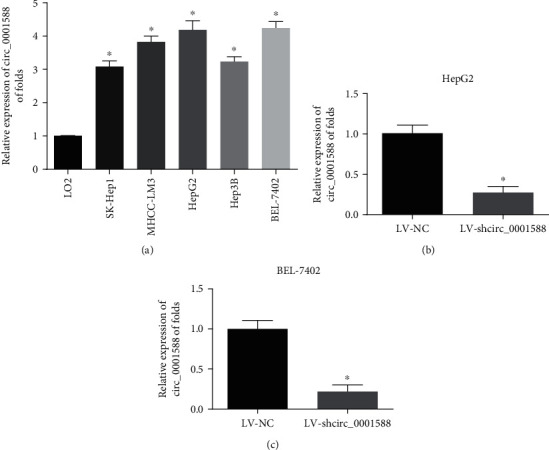
Increase of circ_0001588 expression in HCC cells. (a) Expression of circ_0001588 in HCC cells (SK-Hep-1, Hep-3B, HepG2, BEL-7402, and MHCC-LM3) and LO2 cells. (b, c) The expressions of circ_0001588 were measured in HepG2 and BEL-7402 cells using RT-qPCR. Cells were infected with LV-NC or LV-shcirc_0001588. ^∗^*P* < 0.05.

To identify the expression levels of circ_0001588 in HCC, as shown in Figure 1(a), we observed that the expression of circ_0001588 in HCC cells (SK-Hep-1, Hep-3B, HepG2, BEL-7402, and MHCC-LM3) was also more elevated compared to that in LO2 cells. A potential association between circ_0001588 and HCC was demonstrated. Further, we identified the detailed function of circ_0001588 in HCC cells by using shRNA to silence circ_0001588. As displayed in Figures 1(b) and 1(c), circ_0001588 expression was greatly reduced by circ_0001588 shRNA in HCC cells.

### 3.2. circ_0001588 Exerts a Vital Role in HCC's Oncogenesis

**Figure 2:**
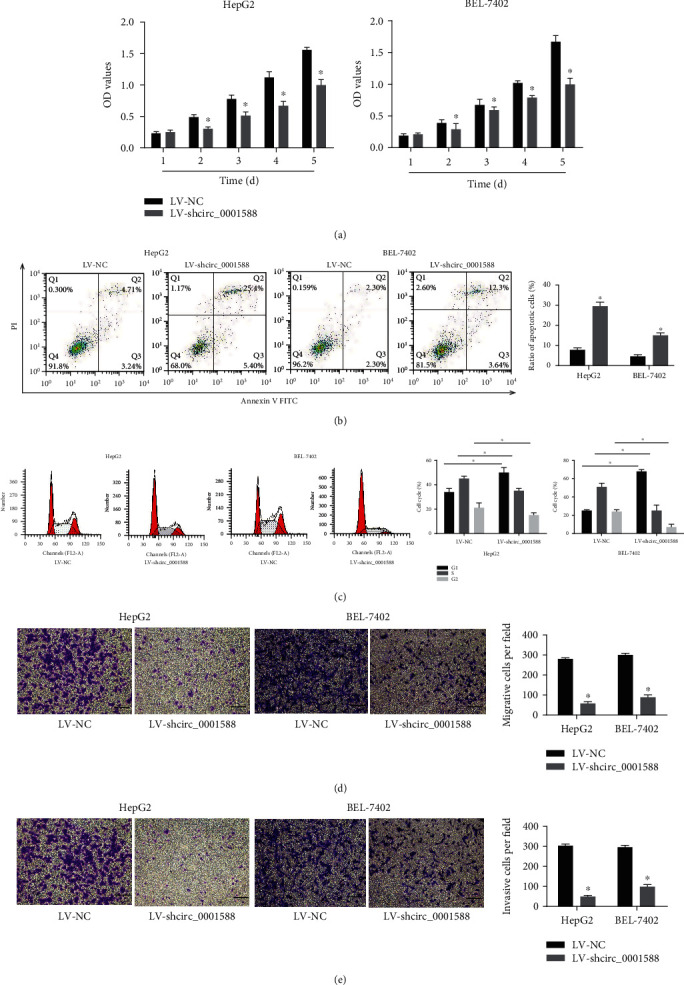
circ_0001588 exerted oncogenic effects on HCC cells. (a) The growth curves were measured by CCK-8 assays. (b) Cell apoptosis was tested using flow cytometry. (c) Cell cycle distribution was assessed using flow cytometry. (d, e) Cell migration and invasion assays were carried out in HCC cells. Scale bar = 100 *μ*m. ^∗^*P* < 0.05.

Proliferation of cells in which circ_0001588 was silenced was analyzed using the CCK-8 test. We found that HCC cell growth was decreased in circ_0001588 shRNA-transfected cells compared to that in the control cells (Figure 2(a)). HCC cell apoptosis was induced by the lack of circ_0001588, while cell cycle progression was blocked by circ_0001588 shRNA (Figures 2(b) and 2(c)). Migration and invasion studies indicated that circ_0001588 downregulation decreased HepG2 and BEL-7402 migration and invasion capacity (Figures 2(d) and 2(e)). The above data indicate the potential role of circ_0001588 in tumorigenesis through the regulation of proliferation, migration, invasion, and apoptosis in HCC cells.

### 3.3. circ_0001588 Promotes the Growth of Liver Cancer Xenograft

**Figure 3:**
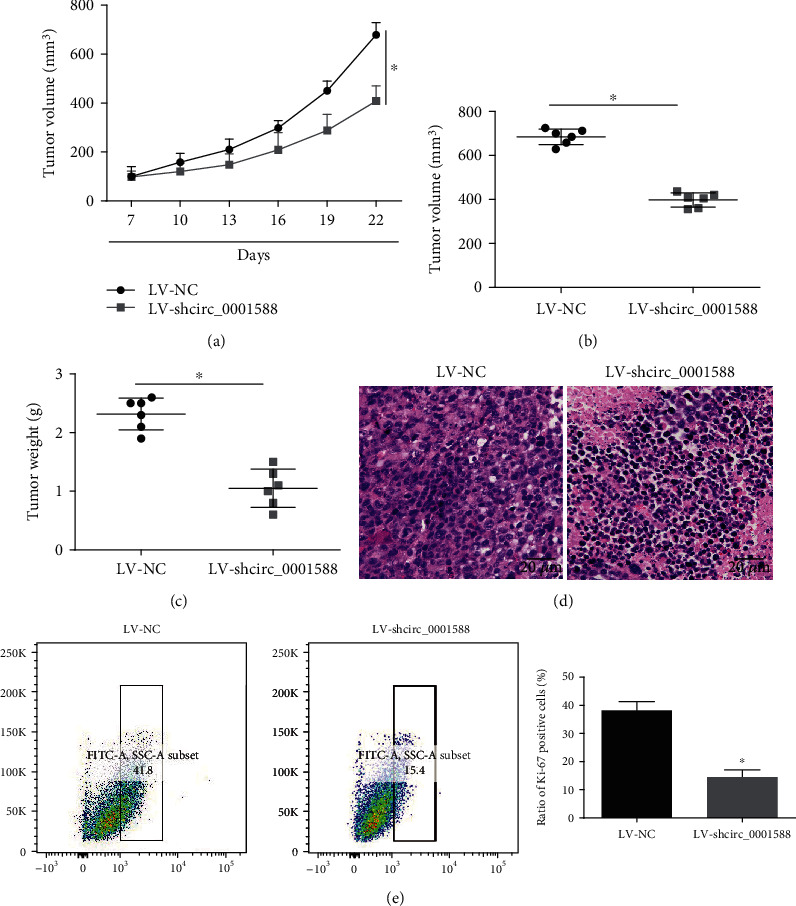
circ_0001588 promoted the growth of liver cancer xenograft. (a) Subcutaneous transplantation of HepG2 cells with silenced circ_0001588 in BALB/c nude mice. Tumor volume was measured every 3 days (volume = width^2^ × length × 1/2). (b) Tumor volume when the mice were sacrificed. (c) After 22 days, the xenograft tumors were excised from the nude mice. The relative weights of tumors were analyzed. (d) HE staining of the xenograft tumor tissues. (e) Ki-67 in the xenograft tumors was examined by flow cytometry. ^∗^*P* < 0.05.

We developed a BALB/c nude mouse model to study the role of circ_0001588 in HCC. Initially, circ_0001588 was inhibited in HepG2 cells transplanted subcutaneously into nude mice. Tumor growth was monitored for 22 days (Figure 3(a)), showing a decrease in the volume of the tumors in the circ_0001588 shRNA group. The tumor volume was significantly reduced by circ_0001588 shRNA (Figure 3(b)). It was evident that the tumor weight decreased significantly in the group of mice with inhibited circ_0001588 (Figure 3(c)). We assessed the tumors isolated 22 days after implantation using H&E and Ki-67 assays (Figures 3(d) and 3(e)), showing that the Ki-67 positive cell ratio was reduced in the circ_0001588 shRNA group, indicating a slower proliferation when compared with sh-NC.

### 3.4. circ_0001588 Targets miR-874

**Figure 4:**
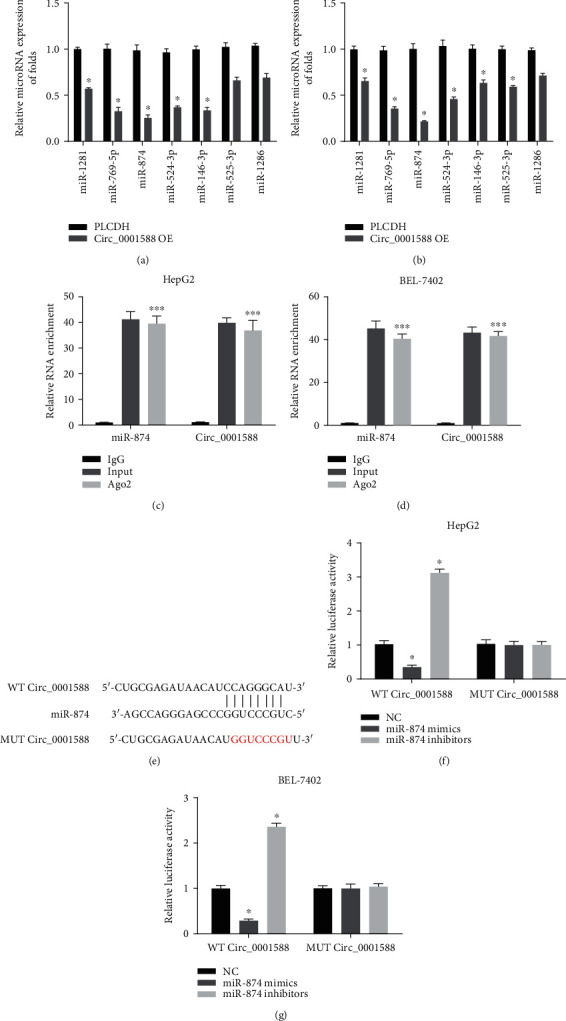
miR-874 was targeted by circ_0001588. (a, b) MicroRNAs regulated by circ_0001588. HCC cells were infected with circ_0001588-OE. (c, d) After lysis, the collected cells were incubated with A/G beads containing Ago2 or the IgG antibody. Levels of miR-874 and circ_0001588 were detected by qRT-PCR. (e) The putative binding sites between miR-874 and circ_0001588 and the mutant sites in circ_0001588-MUT reporter were displayed. (f, g) Luciferase activity was evaluated in HCC cells cotransfected with circ_0001588-MUT or circ_0001588-WT reporter and miR-874 inhibitors or mimics. ^∗^*P* < 0.05; ^∗∗∗^*P* < 0.001.

To further understand circ_0001588 binding targets, we analyzed the circ_0001588 overexpressing cells and found that miR-874 exhibited the lowest expression in the circ_0001588 overexpressing cells (Figures 4(a) and 4(b)). We performed an RNA immunoprecipitation assay with an anti-Argonaute2 (Ago2) antibody to pull down circ_0001588 and miR-874 using anti-Ago2 antibodies or control IgG. We observed that circ_0001588 and miR-874 were significantly pulled down with anti-Ago2 antibodies in HCC cells (Figures 4(c) and 4(d)). Additionally, we performed a luciferase binding assay, wherein labeling vectors were designed for circ_0001588 WT and MUT vectors (Figure 4(e)) with appropriate luciferase labeling. It was evident (Figures 4(f) and 4(g)) that, in the presence of miR-218-5p, in the cells expressing circ_0001588 wild-type, there was a significant decrease in the luciferase activity compared to the control (mimics-NC), while inhibitors exhibited increased luciferase activity.

### 3.5. miR-874 Interacts with and Regulates CDK4 Expression

**Figure 5:**
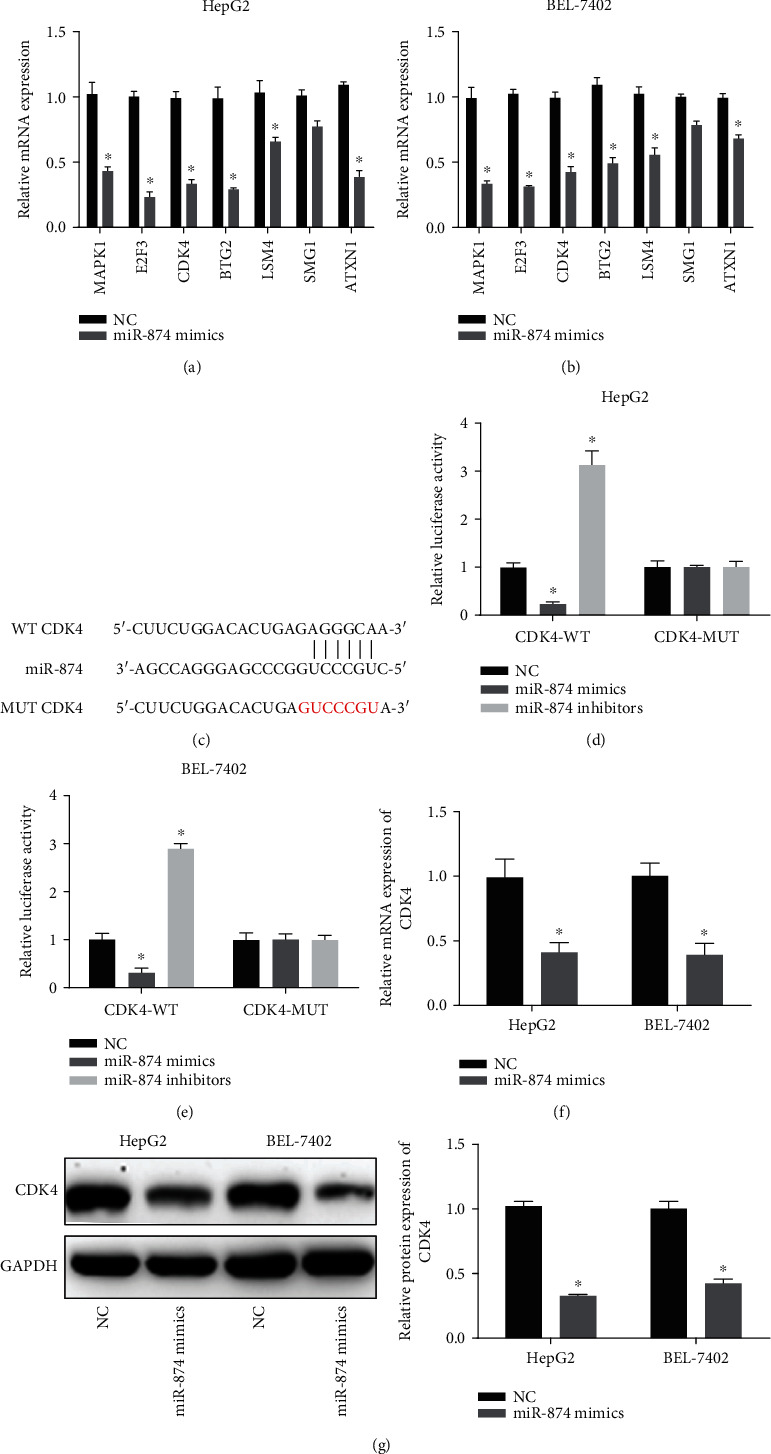
CDK4 was targeted by miR-874. (a, b) mRNAs regulated by miR-874. HCC cells were infected with miR-874 mimics. (c) The putative binding sites between miR-874 and CDK4 and the mutant sites in the CDK4-MUT reporter were displayed. (d, e) Luciferase activity was evaluated in HCC cells cotransfected with CDK4-MUT or CDK4-WT reporter and miR-874 inhibitors or mimics. (f, g) CDK4 expression HCC cells transfected with miR-874 mimics. ^∗^*P* < 0.05.

We found that CDK4 expression was significantly reduced by miR-874 mimics in HCC cells (Figures 5(a) and 5(b)) and identified CDK4 as an important target of miR-874. To assess the binding capacity between them, we designed vectors for the wild-type 3′-UTR of CDK4 and a mutant 3′-UTR of CDK4 with mutations at the predicted miR-874 binding sites (Figure 5(c)). As shown in Figures 5(d) and 5(e), a significant decrease in luciferase activity was observed in cells cotransfected with the wild-type 3′-UTR of CDK4 and miR-874 mimics; there was no decrease in luciferase activity in cells expressing mutant CDK4. CDK4 expression was negatively regulated by miR-874 mimics in HCC cells (Figures 5(f) and 5(g)).

### 3.6. circ_0001588 Promotes the Progression of HCC via Targeting miR-874 and CDK4 Pathway

**Figure 6:**
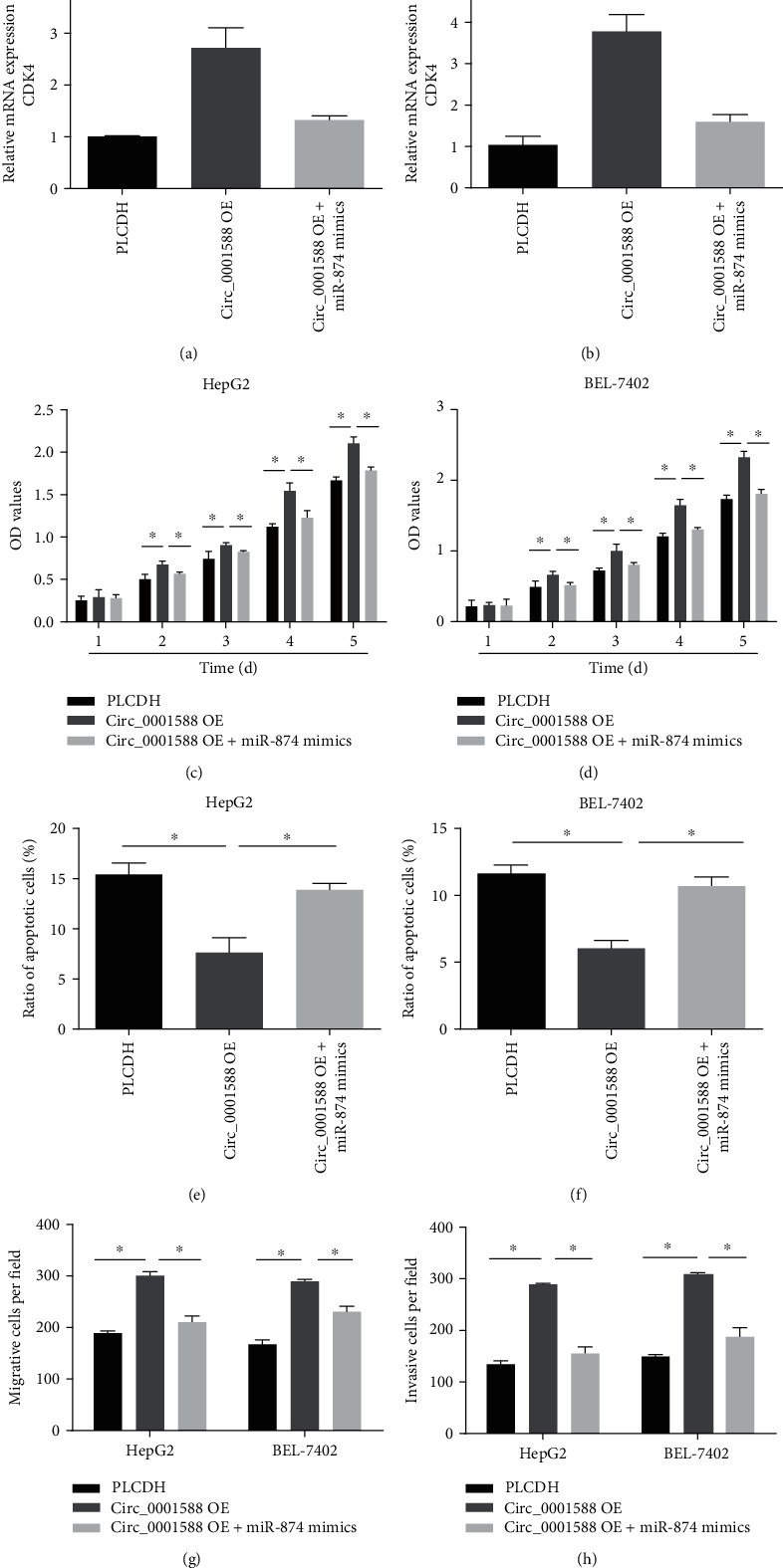
circ_0001588 enhanced the proliferation, migration, and invasion of HCC via targeting the miR-874/CDK4 pathway. (a, b) mRNA expression of CDK4 in HCC cells transfected with circ_0001588-OE and miR-874 mimics. (c, d) The growth curves were tested by CCK-8 assays. (e, f) Cell apoptosis was tested using flow cytometry. (g, h) Cell migration and invasion assays were conducted in HCC cells. ^∗^*P* < 0.05.

To understand the interplay between circ_0001588, miR-874, and CDK4, HCC cells were infected with circ_0001588-OE and miR-874 mimics. As shown in Figures 6(a) and 6(b), we found that circ_0001588 overexpression increased the expression of CDK4 that was reversed by miR-874 mimics in HCC cells. Additionally, we observed that circ_0001588 overexpression enhanced HCC cell proliferation, while overexpression of miR-874 reduced cell proliferation (Figures 6(c) and 6(d)). Apoptosis of HCC cells was inhibited by circ_0001588 overexpression, while miR-874 mimics triggered cell apoptosis (Figures 6(e) and 6(f)). We have shown that induced HCC migration and invasion capacity by circ_0001588 were reversed by the overexpression of miR-874 (Figures 6(g) and 6(h)). This further strengthens our evidence of the significant role of circ_0001588 and miR-874 in HCC progression.

## 4. Discussion

In this study, we observed that circ_0001588 expression was increased in HCC. To the best of our knowledge, this is the first report on elevated circ_0001588 expression in HCC. circ_0001588 induces the progression of lung cancer by modulating miR-524-3p and NACC1 signaling [20]. Here, we found that the loss of circ_0001588 suppressed the malignant behavior of HCC cells. Hence, circ_0001588 may be a therapeutic target for HCC; our data may shed new light on the pathogenesis of HCC.

circRNAs bind to their targeted microRNAs and act as ceRNAs to sponge the corresponding microRNAs and repress their activity [21, 22]. For example, circRNA_0084043 promotes melanoma progression by regulating miR-153-3p and the Snail axis [23]. Dysregulated circ_100876 represses the proliferation of osteosarcoma cancer cells by targeting miR-136 [24]. circMTO1 serves as a sponge for miR-9 to inhibit HCC progression [25]. In our study, miR-874 was predicted to be a target of circ_0001588; miR-874 has been recognized as a tumor suppressor in HCC. For example, miR-874 inhibits HCC proliferation and metastasis by targeting DOR/EGFR/ERK signaling [26]. miR-874 suppresses metastasis and EMT in HCC by targeting SOX12 [27]. We have demonstrated that miR-874 is downregulated in HCC and observed a negative correlation between miR-874 and circ_0001588. Our findings provide new insights into the molecular regulation of circ_0001588 in HCC.

Moreover, miR-874 targets serine-threonine kinase cyclin-dependent kinase 4 (CDK4), a tumor oncogene. CDK4 is a pivotal cell cycle regulator that triggers an important cascade of events in the G1-phase by efficiently catalyzing Rb phosphorylation [28, 29]. The CDK 4/6-inhibitor of CDK4- (INK4-) retinoblastoma (Rb) pathway controls cell cycle progression by regulating the G1-S checkpoint [30, 31]. Palbociclib is a selective CDK4/6 inhibitor that restricts tumor growth in preclinical models of HCC [32]. We have determined the relationship between miR-874 and CDK4 expression in HCC in this study. CDK4 expression in HCC cells was negatively regulated by miR-874; miR-328-5p overexpression inhibited CD4 expression, which was induced by circ_0001588. Future studies should investigate the correlation between circ_0001588 and CDK4.

In summary, we have indicated that circ_0001588 acts as a tumor oncogene to induce HCC progression. We observed that circ_0001588 effectively sponged miR-874 that targeted CDK4. The loss of circ_0001588 significantly reduced HCC tumor growth *in vivo* by regulating miR-874 and CDK4. Hence, the circ_0001588/miR-874/CDK4 pathway may be critical for treating HCC.

## Figures and Tables

**Table 1:** Primers for real-time PCR.

Genes	Forward (5′-3′)	Reverse (5′-3′)
GAPDH	GCACCGTCAAGGCTGAGAAC	TGGTGAAGACGCCAGTGGA
circ_0001588	GGTGTCAAGCGCATTTCTGG	GACGCTTAGCGCCTCCTTTA
CDK4	TGAAATTGGTGTCGGTGCCT	CAGTCGCCTCAGTAAAGCCA
miR-874	GAACTCCACTGTAGCA GAGATGGT	CATTTTTTCCACTCCTCTTCTCTC
U6	CTCGCTTCGGCAGCACA	AACGCTTCACGAATTTGCGT

## Data Availability

The data used to support the findings of this study are included within the article.
